# The effect of COVID-19 on women’s experiences of pregnancy, birth and postpartum in Indonesia: a rapid online survey

**DOI:** 10.1186/s12884-023-05566-w

**Published:** 2023-05-01

**Authors:** Linda McGowan, Andari Astuti, Firdaus Hafidz, Cesa Pratiwi, Vinami Yulian, Elizabeth Hughes, Arum Pratiwi, Emi Nurjasmi Indomo, Yu Fu

**Affiliations:** 1 School of Healthcare, Faculty of Medicine and Health, University of Leeds, Leeds, UK; 2 Midwifery Department, Faculty of Health Sciences, Universitas ‘Aisyiyah Yogyakarta, Yogyakarta, Indonesia; 3 Department of Health Policy and Management, Faculty of Medicine, Public Health, and Nursing, Universitas Gadjah Mada, Yogyakarta, Indonesia; 4 School of Nursing, Faculty of Health Sciences, Universitas Muhammadiyah Surakarta, Jawa Tengah, Indonesia; 5 School of Health and Social Care, Edinburgh Napier University, Edinburgh, UK; 6 Central Board of the Indonesian Midwives Association, Jakarta, Indonesia; 7 Primary Care & Mental Health, University of Liverpool, Liverpool, UK

**Keywords:** COVID-19, Pregnancy, Women's experience, Survey, Indonesia

## Abstract

**Background:**

The interrelationship of psychological and social factors in the current COVID-19 pandemic has been highlighted in research mainly focused on the global north. The impact of lockdowns can exacerbate psychological distress and affect access to services. Less is known about the psychosocial impact on women in the context of lower-middle income countries (LMICs); the aim of this study was to capture the impact of COVID-19 on women’s experiences of pregnancy, birth and postpartum in Indonesia.

**Methods:**

We conducted a rapid cross-sectional online survey of women across all 34 provinces in Indonesia to capture participants’ experiences. Data were collected between 10th July to 9th August 2020 including demographics, effects on general and mental health and impact on service use. Descriptive statistics and thematic analysis were used to analyse responses, including those women who self-identified with a pre-existing mental health problem.

**Results:**

Responses were obtained from 1137 women, this included pregnant women (n = 842) and postpartum women (n = 295). The majority of women (97%) had accessed antenatal care during their pregnancy, but 84% of women reporting feeling fearful and anxious about attending visits, resulting in some women not attending or changing provider. A small number (13%) were denied the presence of a birth companion, with 28% of women reporting that their babies had been removed at birth due to protocols or baby’s health. Feeling anxious was a common experience among women (62%) during their pregnancy, birth or postnatal period, with a small number (9%) feeling depressed. Lockdown measures led to tensions within personal and family relationships.

**Conclusions:**

Women in Indonesia reported that the pandemic added an increased burden in pregnancy, birth and post-partum period: physically, psychologically, spiritually and financially. Maternity services were disrupted and health insurance cover lacked responsiveness, which either directly or indirectly impacted on women’s choices, and equal access to care. Given the longevity of the current pandemic there is a need to develop tailored supportive interventions for women and their families and develop bespoke training for midwives and other relevant health professionals.

**Supplementary Information:**

The online version contains supplementary material available at 10.1186/s12884-023-05566-w.

## Background

The experience of being pregnant, about to give birth, or having a newborn baby is particularly challenging during the coronavirus (COVID-19) pandemic with health services heavily impacted due to lockdowns and restrictions. Health professionals perceived that COVID-19 had directly affected service provision with women being denied birth partners, babies separated from mothers at birth, and having shorter stays during the post-natal period, and compromised care [1]. Evidence also suggests that maternal COVID-19 infection during pregnancy is associated with adverse birth outcomes, such as preterm birth and delivery by caesarean Sect. [2].

The COVID-19 pandemic has had an adverse effect on people’s mental health globally [3]. There are known associations between mental health (anxiety/depression) and poorer pregnancy and birth outcomes (preterm birth; small for dates) [4]. Despite that the fact that maternity services delivered in higher income countries (HICs) were more prepared than lower middle income countries (LMICs) [5], COVID-19 still has impacted populations at a social and psychological level in many HICs. A study in the UK assessed the psychological and social experiences of 600 women with babies postpartum and found elevated rates of depression (43%) and anxiety (61%) as a consequence of social distancing measures. A third of women with mental health issues had not been assessed nor accessed support and treatment [6, 7]. In Canada, a comprehensive survey of 1,987 women revealed elevated symptoms of depression and anxiety during pregnancy (> 35 weeks) as compared with similar cohorts pre pandemic [8]. Symptoms of depression were also exacerbated by deprivation. In the US, women who experienced financial hardship during pregnancy were twice as likely to report symptoms of depression [9].

The World Health Organisation (WHO) is currently collecting clinical data to establish the short and longer term outcomes for COVID-19 during the perinatal period [10]. However, the full impact of the current pandemic on women’s experience and maternity outcomes is as yet not fully understood.

During the context of COVID-19 pandemic, it is more important to assess and manage women’s psychological and social wellbeing during pregnancy, birth and the postpartum period globally. An online survey undertaken early in the pandemic (March/April 2020) with health professionals involved in the delivery of maternity care across 87 countries reported that some facilities were ill-equipped to address the demands because they had limited access to screening, testing, isolation rooms and specific training and guidance for staff [5]. Whilst there is emerging policy and guidance on service provision in LMICs, there is a paucity of research relating to the experiences of pregnant and postpartum women in the current pandemic.

Indonesia is a middle income country with high maternal mortality rates (177 per 100,000, 2017)[11].To improve equity of access to maternal services the government has implemented a National Health Insurance programme covering 222 million people (82%) [12]. Maternity services in Indonesia can be accessed by all, and are provided by primary healthcare centres, maternity clinics, hospitals, private obstetricians and midwives. Prior to the pandemic, women could visit the healthcare centre at any point during pregnancy without making an appointment. This access has been restricted since the pandemic to include extra procedures such as teleregistration, screening for COVID-19 and more reliance on telemedicine [13].

This study aimed to capture the impact of COVID-19 on women’s experiences of pregnancy, birth and postpartum (up to 6 weeks post-delivery) in Indonesia. Two core objectives were to: (i) explore women’s experiences across a range of settings (primary health centres, hospitals, urban and rural areas), and (ii) include women who have diagnosed mental health conditions (pre-existing or pregnancy related) or self-identify as having psychological distress during pregnancy and postpartum.

## Methods

### Study design, sampling and recruitment

The study design is a cross-sectional online survey. The inclusion criteria were women who reported that they were pregnant; had recently given birth; or were postpartum. This included women at all stages of pregnancy, primigravida and multigravida and up to 6 weeks postpartum. A non-probability and convenience sampling method were used to capture and maximise participants ‘experiences and concerns’, due to practical considerations of the accessibility of the population, time and resources available. Two Indonesian research assistants helped to co-ordinate the study, with support provided by the Indonesian Midwives Association (membership of over 400,000 midwives) and MotherHope (an NGO that supports women with perinatal mental health problems) for publicising and distributing the survey across 34 provinces. Prior to giving informed consent, all potential participants were provided with a participant information sheet (PIS) which included the aims of the study, why they had been asked to take part, what was involved and what would be done with their data. All participants provided informed consent online prior to entering the survey site and all respondents were anonymised. The survey was open for four weeks (10th July to 9th August 2020).

### Ethical considerations

Ethical approvals were granted from the relevant ethical committees in both the UK (HREC-19-27, School of Healthcare Research Ethics Committee, dated 7th July 2020) and Indonesia (1629/KEP-UNISA/V/2020, dated 8th May 2020). As this was an online survey, it was not possible to undertake individual welfare checks. All participants were made aware in the PIS that the questions included some on mental health and the PIS included information about where to seek help and support. In addition, participants had the option to contact the two Indonesian researchers (AWA and CSP) that if they needed support and then they could signpost them to relevant resources or the nearest healthcare professional with the women’s consent. It was noted that no women contacted the researchers during the survey period.

### Survey questionnaire

The questions were developed by the study team with expertise in midwifery, applied health research, health psychology, mental health and health systems and management in English then translated to Bahasa Indonesia.

Closed questions with embedded open-ended questions were used to enable women to provide comments on their responses, which led to a mixed methods analysis. Questions addressed the following domains: demographic information including insurance coverage; obstetric history, pregnancy, birth and postnatal experiences; service use in relation to maternal and mental health care and interaction with key health professionals (cadres, midwives, doctors, psychiatrists, psychologists), the effects of COVID-19 on both general and mental health and the impact on service use.

A pilot was conducted with ten respondents from various backgrounds (education, occupancies, and provinces) either ante-natal or post-natal women to ensure clarity and understanding. The results from the pilot were used to refine the survey questions as well as the technical guideline for the survey. The questionnaire is provided (supplementary file 1).

The survey was delivered using Survey Monkey software (*premium version*) (https://www.surveymonkey.co.uk/). The recruitment process involved seeking support from the Central Board of Indonesian Midwives Association to distribute an invitation to take part in the study to 34 of its’ branches (within 34 provinces). In addition, social media platforms (Instagram, Facebook, WhatsApp) were used to invite potential respondents.

### Analysis

The survey was translated back into English prior to analysis. The survey data was analysed using descriptive statistics [14] to assess recruitment, dropout rates and the distribution of characteristics and responses. Chi-square tests were applied to compare categorical variables between women who were pregnant and those in the postpartum period. Where there are rare observations, Fisher exact test was applied. Logistic regression was performed to examine the associations between experienced delay or difficulty in accessing health facilities and respondents’ self-reported anxiety and depression status, adjusted for demographic variables including age, region, education, and occupation status. For all analyses, a two-tailed *p*-value less than 0.05 was considered statistically significant.

Open-ended questions were analysed using a thematic framework [15] facilitated by NVIVO v12 [16]. This involved several processes including stages, familiarisation of the data, generating initial codes, searching for themes, and the refinement and defining of the final thematic chart. The results from the quantitative and qualitative analysis were then examined, discussed and integrated by comparing each participants’ open text with their responses and the distribution of the outcomes to ensure sufficient explanation was provided on responses.

## Results

### Characteristics of the respondents

The total number of responses of the survey were 1613, however, 476 (29%) women provided consent but provided incomplete responses which were removed from the final analysis. Of the 1137 respondents who completed the survey questionnaire, this included both pregnant women (n = 842) and postpartum women (n = 295) (Table 1). The mean age was 28 years old. Respondents were recruited from all 34 provinces across five regions in Indonesia; the majority (84%) were Muslim. 47% held a bachelor’s degree or above, and 48% were housewives. Most of the sample (72%) reported having health insurance coverage for maternity services. The respondents’ education levels and types of employment were not consistent with the general population because not every woman can access the internet. Therefore, people with lower incomes, less education, and living in rural areas may be underrepresented. However, we have tried to capture women across all 34 provinces in Indonesia to demonstrate diversity across geographical locations.

**Table 1:** Characteristics of the respondents (N = 1137)

Characteristics	Pregnant women (N, %)		Postpartum (N, %)		*P* value	All (N, %)	
**Total respondents**	842	74.05	295	25.95		1137	100
**Age** (mean, SD)	28.27	4.80	29.02	4.77	0.030*		
**Age group**					0.642		
< 29	526	62.47	177	60		703	61.83
30–34	223	26.48	80	27.12		303	26.65
>=35	93	11.05	38	12.88		131	11.52
**Regions**					0.052		
Sumatera	132	15.68	53	17.97		185	16.27
Java-Bali	367	43.59	146	49.49		513	45.12
Kalimantan	138	16.39	49	16.60		187	16.45
Sulawesi	84	9.98	20	6.78		104	9.15
Nusa Tenggara-Maluku-Papua	121	14.37	27	9.75		148	13.02
**Religion**					0.049		
Islam	695	82.54	258	87.46		953	83.82
Non-Islam (comprising Christian, Catholic, Hindu and Buddhist)	147	17.46	37	12.54		184	16.18
**Education level**					0.965		
High school and below	252	29.93	87	29.49		339	29.82
Diploma	198	23.52	68	23.05		266	23.39
Higher education	392	46.56	140	47.46		532	46.79
**Occupation**					0.078		
With employment experience	464	55.11	145	49.15		609	53.56
Housewife	378	44.89	150	50.85		528	46.44
**Health insurance for maternal services**					0.433		
No	123	14.61	35	11.86		158	13.90
Yes	595	70.67	219	74.24		814	71.59
Didn’t answer	124	14.73	41	13.90		165	14.51

*Wilcoxon rank-sum test

**Fisher exact test

### Women’s experiences of the pregnancy journey

#### Access to antenatal care

The majority of respondents (97%) had accessed antenatal care (ANC) during their pregnancy, however, ease of access and quality of ANC care varied. A large proportion of women (84%) reported feeling fearful and anxious about attending antenatal visits on one or more occasion due to concerns about the pandemic.

...*worried to get infected, because every time I have an antenatal visit, I took my 2 other children as there is no one could babysit my children, therefore I am afraid of contracting the virus at the healthcare centre*.

The process of both national and regional “lockdowns” in response to the pandemic also affected access to services. Barriers to accessing ANC included facility closures, travel restrictions, and changes to how women could engage with antenatal services for example, having a companion,

“No companion is allowed in the examination room.”

Women also mentioned that booking an antenatal and/or postpartum visit had become more complicated because clinics needed to limit the number of women attending at each session,

…now before (we come) to antenatal booking visit, we have to make a confirmation (appointment) via WhatsApp since the visitors (in the health facility) is restricted everyday.

A third of women (31%) reported that the frequency of antenatal visits was reduced, with a further third reporting that they had been instructed by their provider not to attend ANC visits, unless in an emergency. For some women (27%), services were delivered remotely (mobile phone/online), whilst others chose to move to a clinic that was “*less busy*.” Fear of becoming infected with COVID-19 led some women to avoid, limit or disengage with antenatal services,

Due to the pandemic I am afraid to visit the health centre. Initially I refused to go to the clinic as I live in the red zone and worried I might get infected whilst I am in the clinic.

#### Quality of antenatal care

Just over half the sample (56%) of women noted that the pandemic had resulted in changes to their ANC provision, which had affected the quality of care they received. This led to some women expressing dissatisfaction with services including consultations feeling “rushed”, *“The pregnancy examination went faster”* and not being given enough time to ask questions or raise concerns, *“… the service provided seemed to be in a rush and there were unanswered questions.”* Most women sought information about the effects of COVID-19 on pregnancy and birth (80%), with the majority (75%) stating they had received specific information from healthcare providers. Despite some women having difficulties accessing antenatal services, almost all the women (96%) who had received care rated their experience as ‘very good’ or ‘good’ (Table 2).

**Table 2:** Characteristics of the latest pregnancy (N = 1137)

Characteristics	Pregnant women (N, %)		Postpartum (N, %)		*P* value	All (N, %)	
**Total respondents**	842	100	295	100		1137	100
**Use of antenatal care during the latest pregnancy**					0.126		
No	33	3.92	6	2.03		39	3.43
Yes	809	96.08	289	97.97		1098	96.57
**Feeling fear/anxiety about antenatal visit due to COVID-19**					0.084		
Yes	296	35.15	127	43.05		423	37.20
Sometimes	410	48.69	127	43.05		537	47.23
Rarely	89	10.57	30	10.17		119	10.47
No	47	5.58	11	3.73		58	5.10
**Changes in antenatal care due to COVID-19**							
Yes, reduced frequency on visit	252	29.93	105	35.59	0.071	357	31.40
Yes, health provider suggested not to visit unless emergency	286	33.97	127	43.05	0.005	413	36.32
Yes, I have to change my health provider	39	4.63	8	2.71	0.154	47	4.13
Yes, I contact my health provider through mobile/online	215	25.53	88	29.83	0.151	303	26.65
I prefer to visit a less busy clinic	151	17.93	52	17.63	0.906	203	17.85
I buy vitamin and supplement myself without visiting health provider	77	9.14	26	8.81	0.865	103	9.06
Others	46	5.46	14	4.75	0.635	60	5.28
**Experienced changes in antenatal care due to COVID-19**					0.057		
No	385	45.72	116	39.32		501	44.06
Yes	457	54.28	179	60.68		636	55.94
**Experienced delays or difficulty in accessing health facilities**					0.408		
No	714	84.80	256	86.75		970	85.31
Yes	128	15.2	39	13.22		167	14.69
**Seeking information of COVID-19 on pregnancy during antenatal visit**					0.308		
No	132	15.68	48	16.27		180	15.83
Yes	677	80.4	241	81.69		918	80.74
Didn’t answer	33	3.92	6	2.03		39	3.43
**Received information of COVID-19 on pregnancy by healthcare providers**					0.018		
No	198	23.52	51	17.29		249	21.90
Yes	611	72.57	238	80.68		849	74.67
Didn’t answer	33	3.92	6	2.03		39	3.43

### Preparing for birth

Preparation for birth was affected by the pandemic. A large proportion of women (83%) reported that they had changed their choice of place of birth to a ‘*safer facility’*. For some women this was a deliberate action, for other facilities were either closed or due to COVID-19 were not covered by their health insurance policy (Table 3). Lockdown restrictions meant that women could not choose their birth companion of choice,

I am worried because the virus is everywhere, worried about the preparation for delivery, my parents cannot come to accompany me in the delivery, I am thinking about the delivery procedure, what will happen with my first child when I give birth while the father is working (none will look after my first child).

It was noted that some facilities such as private and public hospitals requested that the childbirth companion should have a proof of COVID-19 negative test in order to be present in the delivery room and/or operating theatre (for caesarean sections).

Of concern is that a small number of women (13%) were denied the presence of a birth companion (husband/relative/friend), with 28% of women reporting that their babies had been removed at birth either due to COVID-19 related protocols or the baby’s health,

*My baby was separated from me because the hospital implements control and prevention of COVID-19.*

**Table 3:** Characteristics of birth (postpartum women only N = 295)

Characteristics	Postpartum (N, %)	
**Total respondents**	295	100
**Whether place of birth changed due to COVID-19**		
No	246	16.61
Yes	49	83.39
**Person accompanied birth process**		
My husband/ close relative/ friend accompanied	232	78.64
My husband/close relative/ friend were not allowed to accompany	39	13.22
I was planning for not having any accompany	2	0.68
Other	22	7.46
**Separated from baby at any time**		
No	213	72.2
Yes	82	27.8

### Effects on psychological wellbeing, personal and social relationships

In Table 2, more than half the sample (62%) reported feeling more anxious/ than usual during the during pregnancy, birth or postnatal period, with a small number (9%) reporting feeling depressed (Table 4). This indicates that anxiety was a common experience among the study population during the pandemic. The circumstances during the COVID-19 pandemic directly impacted on women’s psychological wellbeing. Their main concerns included feeling more worried about the effects of COVID-19 on the baby (42%),

Always worried when going out of house. I am worried that my baby will get infected by the COVID-19 from asymptomatic person (who have been infected by the COVID-19).

Participants described feeling more anxious about family and friends health and general wellbeing (39%), fearful of contracting the disease (38%), and feelings of loneliness and isolation (24%).

I have less support since we are not able to have social meetings due to COVID-19 pandemic situation, I feel lonely. I feel safe during home confinement but I’m lonely.

**Table 4:** Self-reported mental health status (N = 1137)

Characteristics	Pregnant women (N, %)		Postpartum (N, %)		*p-value*	All (N, %)	
**Total respondents**	842		295			1137	100
**Feeling anxious during pregnancy, birth or postnatal**					0.001		
No	296	35.15	140	47.46		436	38.35
Yes	546	64.85	155	52.54		701	61.65
**Feeling depressed during pregnancy, birth or postnatal**					0.948		
No	766	90.97	268	90.85		1034	90.94
Yes	76	9.03	27	9.15		103	9.06
**Whether COVID-19 affected mental health during pregnancy**							
Not at all worried	207	24.58	96	32.54	0.008	303	26.65
I have been worried about getting sick	334	39.67	94	31.86	0.017	428	37.64
I have been worried about my baby	360	42.76	117	39.66	0.354	477	41.95
I have been worried about family and friends	341	40.50	97	32.88	0.021	438	38.52
I have been lonelier and more isolated	193	22.92	75	25.42	0.384	268	23.57
I have not been able to care for others	86	10.21	33	11.19	0.639	119	10.47

Lockdown measures such as social distancing and travel restrictions led to tensions within personal and family relationships,

My husband works in another city and he once couldn’t go home due to the lockdown. So, we had three months of long-distance relationship, (it made our) communication messed up, so many missed understandings. (I think) the main cause was my husband and I were all stress and (we were) anxious about the pandemic.

Living arrangements were changed to suit the need to isolate with some women living with in laws during pregnancy and birth which could lead to potential feelings of isolation and conflict,

I don’t feel safe during home confinement, due to risk of infection because my house is cramped and my in-laws don’t keep it clean…because my husband got transferred and I have to stay at my in-laws house during this pregnancy. During home confinement, I can’t do activities outside normally and that make it easier to have avoid conflict with my family, especially from my husband side. Besides, my pregnancy makes me emotionally sensitive.

All women in the survey were provided with information about the mental health support available in Indonesia. Women were given the option to disclose the presence of existing mental health problems. For those women who revealed they had mental health difficulties, the pandemic situation appeared to heighten their distress,

*As I am a postpartum depression survivor (in the previous childbirth), therefore in this pandemic situation I feel more anxious than usual”* with some describing suicidal ideation, *“I live (at home) alone, my first child is taken by my husband to work as a motorcycle taxi driver. I always think about ending my life*.

Compared to respondents aged 35 and over, women with a younger age were likely to experience anxiety. Respondents with an education background beyond high school and those with no experience of being employed at all were more likely to be anxious. More importantly, women were likely to report being anxious and depressed if they had experienced delays and/or difficulties in accessing health facilities (Tables 5 and 6).

**Table 5:** Logistic regression analysis of self-reported anxiety and demographic data (N = 1137)

Demographic variables		Odds ratio	95% Confidence interval	*p value*
**Age group**	< 29	1.00		
	30–34	1.01	(0.75–1.35)	0.971
	>=35	0.63	(0.43–0.94)	0.023
**Region**	Sumatera	1.97	(1.25–3.09)	0.003
	Java-Bali	1.99	(1.35–2.94)	0.001
	Kalimantan	1.50	(0.96–2.34)	0.078
	Sulawesi	2.20	(1.28–3.79)	0.005
	Nusa Tenggara-Maluku-Papua	1.00		
**Education**	High school and below	1.00		
	Diploma	1.92	(1.31–2.81)	0.001
	Higher education	1.87	(1.33–2.61)	< 0.001
**Occupation**	With employment experience	1.00		
	Housewife	1.31	(0.98–1.76)	0.071
**Experienced delays/difficulty in accessing health facilities**	No	1.00		
	Yes	2.32	(1.57–3.45)	< 0.001

**Table 6:** Logistic regression analysis of self-reported depression and demographic data (N = 1137)

Demographic variables		Odds ratio	95% Confidence interval	*p value*
**Age group**	< 29	1.00		
	30–34	0.97	(0.58–1.63)	0.917
	>=35	0.78	(0.37–1.66)	0.519
**Region**	Sumatera	6.18	(1.76–21.73)	0.005
	Java-Bali	5.39	(1.6-18.22)	0.007
	Kalimantan	5.70	(1.59–20.37)	0.007
	Sulawesi	8.75	(2.44–31.38)	0.001
	Nusa Tenggara-Maluku-Papua	1.00		
**Education**	High school and below	1.00		
	Diploma	0.94	(0.49–1.79)	0.847
	Higher education	0.91	(0.53–1.56)	0.719
**Occupation**	With employment experience	1.00		
	Housewife	1.45	(0.9–2.36)	0.130
**Experienced delays/difficulty in accessing health facilities**	No	1.00		
	Yes	4.72	(3.01–7.4)	< 0.001

## Discussion

This exploratory study captured the immediate impact of the COVID-19 pandemic (and resultant changes to health services delivery and social distancing measures) on Indonesian women’s experiences of pregnancy, birth and postpartum period during July/August 2020. Whilst there is a growing body of research relating to the experience of healthcare professionals and the effects on services, women’s views and experiences remain relatively under studied. We sought to capture women’s experiences via the use of an online survey involving 1137 women across 34 provinces in Indonesia. In addition, we included women who had a diagnosed mental health condition (pre-existing or pregnancy related) or self-identified as having psychological distress during pregnancy and postpartum to explore the effects on mental health on this vulnerable group.

### Women’s experiences of maternity care

Women reported that the pandemic added an increased burden in pregnancy, birth and post-partum period: physically, psychologically, spiritually and financially.The WHO recommended that women should have at least eight antenatal contacts during pregnancy and that services should be improved to facilitate a ‘positive pregnancy experience’ [17]. However, the participants of this survey reported that normal contact with maternity services was severely disrupted and not in line with WHO guidance. At the start of the COVD 19 pandemic, the Indonesian Government initially recommended a minimum of 4 ANC visits for uncomplicated pregnancies, however, in light of the scale of COVID-19 infection that emerged the government thenrecommended the postponing of visits in the second trimester [18], In addition to restriction of appointments the participants reported that they chose not to attend services out of fear of becoming infected. This was particularly noticeable in the antenatal period when some women reported avoiding ANC altogether, whilst some delayed until when they perceived it safe to attend clinics. In line with other research, women described their dissatisfaction with care provision, especially with the requirement to attend ANC appointments and even birth without a companion or partner. In addition, approximately a third of postpartum women were separated from their babies immediately post birth where COVID-19 was suspected, which was not part of national guidance. This disappointment in maternity care services was mirrored in a recent UK survey co-produced with maternity service users and partners [19] in which some users felt lost and let down by the system during the COVID-19 pandemic and that virtual contact did not equate to in-person contact. This suggests that hybrid models are needed in an ongoing pandemic situation [20].

### Disruption of services

The main finding revealed that the pandemic disrupts health systems at macro level, which in turn affects service delivery to women at the meso, and micro level. The lack of preparedness and response to the COVID-19 pandemic of the health system in Indonesia is in line with the findings of [5] a global online survey of 714 maternal and neonatal health professionals. The need for the rapid distribution of resources to deal with the pandemic impacts the ability of maternity care staff to deliver services and the experiences of service users in receipt of care. In Indonesia, several factors affected access to services including disruption of assessments and AN classes for pregnant women, and unprepared services (in terms of personnel and infrastructure) [21], Changes to service delivery were ad hoc and lacked consistency. This impacted directly on women’s access to ANC with less appointments, remote delivery of care via phone/online, lack of choice (due to facility closures) and some women were asked not to attend ANC unless it was an emergency. Our findings resonate in part with the findings from a survey of 226 Indonesian pregnant women in South Sulawesi, who noted that the predictors of the uptake of ANC were women’s own visiting behaviour and access to services [22].

### Effects on psychological wellbeing, personal and social relationships

More than half the respondents in this study reported feeling anxious or more anxious than usual during the perinatal period (62%), whilst only 9% reported feeling depressed. It should be noted that this is a self-reported experience and validated psychological measures were not utilised in this study. However, our findings are supported by a recent cross-sectional study in Indonesia of 120 women to assess psychological outcomes during the current pandemic [23] in which the Depression, Anxiety and Stress Scale-21 (DASS-21) was administered as an online survey for pregnant women or women who had recently given birth. Similar to this study, they reported that just over half the sample had anxiety (53.3%). Our survey further highlights that younger women, with an education attainment higher than high school and those with no experience of being employed, were more likely to self-report feelings of anxiety. These findings are in line with Mutahi et al., (2022) who found that younger pregnant women in Sub-Saharan Africa were more likely to have mental health problems [24]. In Indonesia, research has shown that anxiety was more commonly reported during the pandemic by pregnant women with higher educational levels [23]. In the current study it was noted that women were likely to report being anxious and depressed if they had experienced delays and/or difficulties in accessing health facilities. Future research is needed to continually monitor the psychological health of pregnant and postpartum women in order to tailor support and provide appropriate psychological care for those women in most need.

Social support can reduce levels of anxiety and stress and support individuals during the experience of key life events, such as pregnancy and childbirth [25] however the social isolation imposed by lockdowns and social distancing measures disrupts and disturbs the usual social support networks that women draw on during pregnancy, childbirth and postpartum. In addition to impact of social isolation, the pandemic also added fear of infection and subsequent risks to mother and baby as well as reduced face to face healthcare [8]. In Indonesia, women were especially isolated in lockdown as many men work outside their local area within Indonesian culture, and were forced to stay away due to travel restrictions [26]. In addition, the travel restrictions also prevented family members from visiting. In Indonesian culture the family are a strong source of support; helping new mothers through their maternal transition, as well as practical help household chores, cooking and and childcare [27].

### Health inequalities

The COVID-19 pandemic has deepened existing health inequalities with International Monetary Fund (IMF) noting that ‘COVID-19 is not an equal opportunity virus’ [28]. Changes in service provision could result in increased maternal morbidity and mortality and neonatal complications [29], particularly among people with disadvantaged backgrounds, for example, pregnant women who could not afford private care. More affluent participants reported preferring to access private clinics as they were perceived to be lower risk for COVID as they would be less crowded. Ongoing effects from the pandemic are likely to exacerbate these challenges therefore widening health and social inequalities, with limited monitoring practices and policies implemented to improve equitable care [30], future research is needed to tackle health inequalities experienced by women in this cohort.

### Limitations

This survey was opportunistic and we are unable to fully assess response rates. The women who responded needed to be literate and have access to the internet thus there is a potential to exclude lower socioeconomic, poorly educated women. A different method of data collection would need to be utilised (such as face to face interviews) which was not possible or feasible for this study. There is the possibility of selection bias in who chose to participate; that is, people who had a challenging experience may be more likely to want to take part. This in turn might impact on the generalisability of the findings. However, much of the findings resonate with womens’ experiences of maternity care in other countries and settings. The survey only provides information about women’s self-reported experiences at one time point in time during the COVID-19pandemic, however it does represent a period of time which was characterised by services in transition and a lack of information on best practice in healthcare delivery in a pandemic. It was also at a time prior to the COVID-19 vaccine where fear of infection would be heightened.We relied on self-report of mental health problems. It was beyond the scope of the study to independenly verify the mental health conditions that the respondents reported. A, Despite these limitations, this study collected responses from women who lived in all provinces in Indonesia contributing to the generalisability. It is likely that respondents with biases selected themselves into the sample, however, open texts were available to provide rationales for this response.

## Conclusion

The COVID-19 pandemic impacted directly on the maternity experiences of women in Indonesia, especially in terms of increasing anxiety, loss of social support, and disruption to maternity care services. In addition to strengthening maternity services, there is a need for responsive mental health services for women during this time of potential increased vulnerability. The development of tailored training packages for midwives, and other key health workers, is needed to understand the impact of the current pandemic and the potential longer-term effects on women and families.

## Electronic supplementary material

Below is the link to the electronic supplementary material.

**Additional file 1**.

## Data Availability

The datasets used and/or analysed during the current study available from the corresponding author on reasonable request.
