# Supplementary material for: The effect of COVID-19 on women’s experiences of pregnancy, birth and postpartum in Indonesia: a rapid online survey
Source: BMC Pregnancy Childbirth. 2023 May 1;23:304. doi: 10.1186/s12884-023-05566-w (PMC10150340; doi:10.1186/s12884-023-05566-w)
Supplement: Supplementary file 1 — Additional file 1. [file 12884_2023_5566_MOESM1_ESM.docx]

**The effect of COVID-19 on women’s experiences of pregnancy, birth and postpartum in Indonesia: A rapid online survey**

This is an open survey distributed across 34 provinces in Indonesia. If you are a woman (over 16 years old) living in Indonesia who is currently pregnant, about to give birth or are in the immediate postpartum period (up to six weeks post birth), we would like to hear your views on how the COVID-19 pandemic has affected your experiences and your quality of life.

**Title of study**:

Exploring the effect of COVID-19 on women’s experiences of pregnancy, birth and postpartum in Indonesia: A rapid online survey

**Purpose**:

The purpose of this study is to explore the impact of responses to COVID-19 on women’s experiences of pregnancy, birth and postpartum (up to 6 weeks post birth) in Indonesia.

**Procedures**:

If you decide to participate in this study, you will be asked about your age, education, income, marital status, occupation, employment status, the birth, experiences of antenatal care, interaction with health professionals, and how you have been feeling during the pandemic.

**Confidentiality**:

Your personal information will be kept confidential and securely stored on the University server. You will not be identified in any report or publication. We will produce a short summary of the findings for you by email or on WhatsApp or other social media, on the completion of the study.

**Your rights:**

Your participation in this study is completely voluntary. You can withdraw from this study without giving a reason within two weeks after you have completed the survey. Please contact the researcher to withdraw, this will not affect your health and social care in any way. To be able to trace your information entered in the survey, please enter the last four digits of your mobile before you start the survey.

**Benefits and risks**

The information we collect from this study is unlikely to benefit you directly, however, a summary of the study outcome will be shared with you that may help you understand the effect of COVID-19 on women’s experiences. Taking part will allow your views and experiences to be heard and the information may help improve future services and support for women.

We do not anticipate any risks if you take part. You do not have to answer any questions you do not wish to, and you may pause or stop the survey at any time. If you feel distressed by completing the survey, please discuss any concerns or worries with the researcher who will advise you to contact your local health providers and midwives, and also provide a list of appropriate resources and signposting to relevant services, which will include MotherHope who have access to a number of psychologists and trained volunteers.

**Name and contact details of the lead researchers**

Dr Andari Wuri Astuti, Lecture in UNISA Yogyakarta

Tel: +62 877 363 51159; Email: [astutiandari@unisayogya.ac.id](mailto:astutiandari@unisayogya.ac.id)

Dr Cesa Septiana Pratiwi, Lecture in UNISA Yogyakarta

Tel: +62 822 278 65471; Email: [cesaseptianapratiwi@unisayogya.ac.id](mailto:cesaseptianapratiwi@unisayogya.ac.id)

Before you decide to participate in this study, please confirm that you have read the information sheet (HERE IS THE INFORMATION SHEET) – BOX TO CHECK YES IF NO- EXIT THE SURVEY.

Please confirm the following criteria to complete the survey– ALL BOXES TO CHECK YES IF NO- EXIT THE SURVEY.

Resident of Indonesia and currently pregnant or given birth within the last six weeks from today’s date. Please answer the questions below by choosing one of the closest conditions similar to your situation. For those who have given birth and in the postpartum period, please also fill the survey on pregnancy and childbirth section.

**AGREEMENT TO PARTICIPATE**Please indicate your agreement to participate below:

I Agree

I Do Not Agree

To enable your free withdrawal within two weeks after the survey, please enter the last four digits of your mobile here_____________. This information will be stored securely.

**Compensation**:

As thank you gift for your contribution, 100 participants (will be selected randomly) will be given a 100,000 IDR mobile top up voucher. If you wish to join into this prize win, please provide your mobile number here: …………….. Your data will be stored safely and separately to the survey data.

**QUESTIONS ABOUT YOU**

Age:

Province: (Please tick the box which applies to you)

|  | Nangroe Aceh Darussalam |  | DKI Jakarta |  | Gorontalo |
| --- | --- | --- | --- | --- | --- |
|  | Sumatera Utara |  | Jawa Tengah |  | Sulawesi Utara |
|  | Sumatera Barat |  | DI Yogyakarta |  | Sulawesi Barat |
|  | Riau |  | Jawa Timur |  | Sulawesi Tengah |
|  | Kepulauan Riau |  | Bali |  | Sulawesi Selatan |
|  | Jambi |  | Nusa Tenggara Barat |  | Sulawesi Tenggara |
|  | Bengkulu |  | Nusa Tenggara Timur |  | Maluku Utara |
|  | Sumatera Selatan |  | Kalimantan Utara |  | Maluku |
|  | Kepulauan Bangka Belitung |  | Kalimantan Barat |  | Papua Barat |
|  | Lampung |  | Kalimantan Tengah |  | Papua |
|  | Banten |  | Kalimantan Selatan |  |  |
|  | Jawa Barat |  | Kalimantan Timur |  |  |

Religion :

Moslem

Christian

Catholic

Hinduism

Buddhism

Confucianism

Education :

Primary School

Junior high school

Senior high school

Diploma

Bachelor

Master

Doctoral

Did not attend the school

Employment (you can choose more than one):

Housewife

Civil servant (please specify)……….

Private sector (please specify)……….

Entrepreneur (please specify)……….

State enterprise staff (please specify)……….

Others (please specify)……….

What is the type of national insurance scheme you have?

JKN subsidised scheme

JKN, non-subsidised scheme

Private insurance

Insurance from office/ company

Do not have insurance

If you have health insurance, do/ did you use the health insurance for your maternal healthcare service?

Yes

No

(Please specify the reason for not using the health insurance for your maternal healthcare service)….

Monthly family income:

< Rp 1.000.000

Rp 1.000.000 – Rp 2.499.000

Rp 2.500.000 – Rp 5.000.000

>Rp 5.000.000

What sources of information on COVID-19 and pregnancy, childbirth, postnatal have you used? (You can choose more than one)

Radio

Television

Newspaper or magazine

Family and friends

Website

Social media

Others (please specify) ………

Have you been tested in related to COVID-19?

No/ Never because I believe that I will not contract the disease

No/ never because I am in fear of the result

No/ never because I don’t have the opportunity

No/ never because I don’t have a financial budget

Yes, rapid test

Yes, swab test /PCR

If you have been tested and willing to share the results, please choose from the following options the most applicable

Non-reactive for rapid test

Reactive for rapid test

Negative for swab test /PCR

Positive for swab test /PCR

**QUESTIONS ABOUT YOUR PREGNANCY**

Gravidity :

Number of living children :

Gestational age : (skip for those who have given birth)

The first date of last period :

During pregnancy did/do go for antenatal visit?

Yes

No

If no, why not? ……………

If yes, where do you go for your antenatal visit? (You can choose more than one)…………….

Public hospital

Private hospital

Primary clinic

Maternity clinic

Midwife Private Practice

Home visit (Midwives/ doctors/ healthcare professionals)

Online consultation with healthcare professionals

Other (please specifiy)………….

Who is your main health provider for antenatal care?

Midwife

Doctor

Obstetrician

Traditional birth attendant

Others, please specify ……………..

Have you/did you experience delays or difficulty in reaching the facility / place of ANC contact?

Yes

No

If yes, please indicate the difficulties from the following options (you can choose more than one)

You did not want to go

Your husband or family did not want you to go

You were concerned about getting COVID-19

You have no transport to reach the clinic/ hospital

O Others, please specify ……………..

During the current situation of the COVID-19, do/did you experience any changes in terms of antenatal care or visit?

No, everything runs as planned

Yes

If you choose yes, what are changes that you have experienced during the antenatal visit? (You may choose more than one option)

Frequency on suggested antenatal visit was reduced

My providers have suggested me not to visit the clinic unless there is an emergency (due to risk of infection)

I have to change to another healthcare provider

I can contact and have consultation with my healthcare provider through mobile phone/ online

I prefer to visit a less-busy-clinic

I prefer to buy pregnancy vitamin and supplement by myself without visiting healthcare provider

Other (please specify):…

Did/do you get any information regarding COVID-19 related pregnancy from healthcare providers?

Yes, please specify the information:….

No

If no, what information do you expect you can get from health providers?

Were you able to ask questions about COVID-19 and pregnancy during your antenatal visit?

Yes

No

Did/do you feel fear or anxiety about visiting ANC centre due to the current situation of the COVID-19 pandemic?

Yes, I feel very anxious/ fear of contracting the disease if I visit the healthcare centre

Sometimes, I feel anxious/ fear when I visit the healthcare centre

I rarely feel anxious/ fear to get infected by the disease when visiting the healthcare centre

No, I don’t feel anxious/ fear at all to go to the healthcare centre due to the pandemic

Did you feel that you have less support during your pregnancy due to the current situation of the COVID-19 pandemic?

No, everything runs as normal/ usual

Yes, specify in a short narrative………..

Please tick as applicable, the COVID -19 health and safety protocols that are implemented in the clinic during your antenatal visit: (you can choose more than one option)

Body temperature of the visitor is measured using a thermogun

On arrival all visitors have to wash hand using soap or cleanse using hand sanitizer

Only one companion is allowed to enter the clinic

All visitors have to wear a mask

All visitors have to maintain physical distancing for minimum of one meter

Children are not allowed to enter the clinic

A Asked to attend alone without other family members

Other, Please specify:…..

How was your experience with them (healthcare providers) during antenatal care?

Very good

Good

Bad

Very bad

**QUESTIONS ABOUT THE BIRTH OF YOUR CHILD**

Date of childbirth :

Number of the baby :

Sex of the baby

Girl

Boy

Time/ term of delivery:

Early term (<36 weeks)

Full term (36-42 weeks)

Post-term (>42 weeks)

Place of childbirth

Public hospital

Private hospital

Primary clinic

Maternity clinic

Midwife Private Practice

Home birth

Others (please specify)………….

Did the place of birth change from the planned place due to the COVID-19 pandemic?

Yes, Please mention your changed plan:…

No

Please tick your experience regarding your situation related to the person whom accompanied you during the birthing process

My husband/ close relative/ friend accompanied me during the birthing process

My husband/close relative/ friend were not allowed to accompanied me during the birthing process due to COVID-19 protocol

I was planning on not having anybody to accompany my birthing process

Other situation, please write…..

Who was your main health provider during delivery?

Midwife

Doctor

Obstetrician

Traditional birth attendant

Others, please specify

Mode of delivery:

Normal delivery without any intervention

Normal delivery with intervention (e.g. induction, vacuum, forceps, etc)

Caesarean section

Were you separated from your baby at any time?

Yes (please specify the reason………….)

No

Did you feel that you had less support during your childbirth due to the current situation of the COVID-19 pandemic?

No, everything run as normal/ usual

Yes

please specify in a short narrative………..

Condition of newborn:

Healthy

Compromised

If the baby had a compromised condition/ complication which caused the baby to be moved to intensive care or referral treatment, please tick as applicable

Difficult to breathe

Low birth weight (less than 2500gram)

Preterm baby (born before 36 weeks)

Post-term baby (born after 42 weeks)

Congenital malformation

Other, please specify the compromised condition/ complication:…….

If there was a compromised condition, did the baby receive intensive care treatment?

Yes

No

If the baby received intensive care treatment, write down the specific care:….

If the baby did not receive intensive care, write down the reason:….

How was your experience with them (healthcare providers) during the childbirth?

Very good

Good

Bad

Very bad

**QUESTIONS ABOUT YOUR POST-NATAL PERIOD**

Where did/do you access health support during the postnatal period?

Public hospital

Private hospital

Primary clinic

Maternity clinic

Midwife Private Practice

Online consultation

Home (midwife/ doctor/ healthcare professionals visit your home)

Others (please specify)………….

During the COVID-19 pandemic, did you experience changes in terms of access to maternity care during the postnatal period that you received?

No, everything runs as I and my provider planned

Yes

Please specify the difference in a short narrative………..

During the COVID-19 pandemic, did you experience changes in terms of seeking help during the postnatal period?

No, everything runs as I and my provider planned

Yes

Please specify the difference in a short narrative………..

Have/do you feel fearful or anxious to visit the health care centre for postnatal check due to the current situation of the COVID pandemic?

Yes, I feel very anxious/ fearful of contracting the disease if I visit the healthcare centre

Sometimes, I feel anxious/ fearful when I visit the healthcare centre

I rarely feel anxious/ fearful about getting infected by the disease when visiting the healthcare centre

No, I do not feel anxious/ fearful at all to go to the healthcare centre due to the pandemic

Do/ did you feel that you have less support during the postnatal period due to the current situation of the COVID-19 pandemic?

No, everything runs as normal/ usual

Yes

Please specify in a short narrative………..

Please tick as applicable the health and safety protocols that are implemented in the clinic during your postnatal visits: (you can choose more than one)

Body temperature of the visitor is measured using a thermogun

All visitors have to wash hand using soap or cleanse using hand sanitizer

Only one companion is allowed to enter the clinic

All visitors have to wear a mask

All visitors have to maintain physical distancing for minimum of one meter

Children are not allowed to enter the clinic

Other, please specify: …..

Is the baby breastfed?

Yes, just breastmilk

Yes, breast milk and formula

Formula only

Why do you choose formula to feed your baby?

I am afraid my baby might get infected by COVID-19 -19

I do not produce adequate breastmilk

I had breast engorgement

I had cracked nipples / problem with my nipples

Others, please specify:………

Do your baby get vaccinated?

Yes

No

If you choose no, what is your reason? (You may choose more than one option)

Fear of visiting clinic due to the current situation of the COVID-19 pandemic)

Fear of the side effects of immunization

Against my religious beliefs

Against my personal beliefs

Other: ………………..

Where did your baby receive their vaccination? (You can choose more than one)

Private hospital

Public hospital

Primary clinic

Maternity clinic

Midwife Private Practice

Home (midwife/ doctor/ healthcare professionals visit your home)

Others (please specify) ……

Have you received any information regarding contraception and family planning from your healthcare provider?

Yes

No

Please choose the following options based on your situation regarding contraception (you can choose more than one)

I am not using any contraception but I am planning to use one of them

I am not using contraception and I am not planning to use one of them

I use modern contraception (IUD, Implant, Pill, Injection)

I use traditional/ own strategy contraception (lactational amenorrhea method, calculating period, coitus interruptus)

I am having/have a tubectomy / my husband has vasectomy

My husband uses condoms

Other situation, please specify:…..

Where did/do you receive your contraception service?

Private hospital

Public hospital

Primary clinic

Maternity clinic

Midwife Private Practice

Drug store

Cadre

Family planning officer

Other (please specify)………

How is your experience of them (health providers) during your postnatal care?

Very good

Good

Bad

Very bad

**QUESTIONS ABOUT YOUR MENTAL HEALTH AND WELLBEING**

Have you felt anxious during your pregnancy, during birth or postnatal?

Yes

No

If yes, is this:

Same as usual

More than usual

Less than usual

Comments box

Have you felt depressed during your pregnancy, during birth or in the postnatal period?

Yes

No

If yes, is this:

Same as usual

More than usual

Less than usual

Comments box

How do you think the COVID-19 -19 pandemic has affected your mental wellbeing during pregnancy? (You can choose more than one)

I have been worried about getting ill or dying

I have been worried about my baby getting sick/dying

I have been worried about family and friends getting sick/dying

I have been more lonely and isolated in my pregnancy/birth due to COVID-19 -19 restrictions

I have not been able to care for others due to concerns of infection

I have not been affected by COVID-19 -19 in any way

What has helped you cope with being pregnant/giving birth during the COVID-19 -19 pandemic?

Emotional support from family/ relative/ friends/ healthcare professionals

Practical support from family/ relative/ friends/ healthcare professionals (e.g. accommodation, providing food, childcare, household chores, financial, etc)

Information from family/ relative/ friends/ healthcare professionals

Others, please specify…..

Who is your main coping source for being pregnant/giving birth during the COVID-19 -19 pandemic?

Partner

Family members

Friends /relatives

Healthcare professionals

Others, (please specify……..)

Do you have an existing mental health condition (one that existed prior to this pregnancy)?

No

Yes

Are you happy to disclose what this condition is?.............

Have you received any of the following treatments for mental health issues?

Counselling or other talking therapy

Medication

Mental health related hospital admission

No treatment of any kind

If you are receiving mental health treatment – how has this been affected by the COVID-19 -19 pandemic?

Please write down in this box……………….

Have you felt unsafe at any time during home confinement?

No

Yes, due to risk of infection

Yes, due to risk of violence

Comments box

Overall, how was your experience of health providers?

Very good

Good

Bad

Very bad

Is there anything we have missed in this survey that you would like to tell us?

Comments box

Finally, if you have any other things to add about your experience of pregnancy and birth during the COVID-19 -19 pandemic, please use this space here

The COVID-19 -19 pandemic has affected everyone’s lives including mental wellbeing. We want to finally ask you a couple of questions about how you found filling in this survey and to offer advice as to where to seek further support.

1. I was comfortable completing the survey

Very comfortable

Comfortable

Neutral

Uncomfortable

Very uncomfortable

1. The survey was easy to use

Very easy

Easy

Neutral

Difficult

Very difficult

1. I didn’t feel anxious about filling it

Not at all anxious

A little anxious

Moderately anxious

Very anxious

Extremely anxious

For mental health worries – please talk to your health provider or contact Motherhope Indonesia either through Facebook Group https://www.facebook.com/groups/pedulikesjiwaibuperinatalindo/?ref=share or Instagram @motherhopeind

For COVID-19 advice, please see:

https://www.kemkes.go.id/article/view/20012900002/Kesiapsiagaan-menghadapi-Infeksi-Novel-Coronavirus.html

Thank you for taking the time to complete this survey.

The fact that you are reading this message indicates that you have completed our questionnaire and to that we owe you a debt of thanks.

We are very appreciative of the time you have taken to assist in our analysis, and commit to utilizing the information gained to contemplate and implement
worthwhile improvements.

Once again, we are extremely grateful for contributing your valuable time, your honest information, and your thoughtful suggestions.

Finally, we hope all the best for your health and wellbeing.
